# Benefit of serum drug monitoring complementing urine analysis to assess adherence to antihypertensive drugs in first-line therapy

**DOI:** 10.1371/journal.pone.0237383

**Published:** 2020-08-10

**Authors:** Sabrina Ritscher, Milena Hoyer, Coralie Georges, Cora Wunder, Pierre Wallemacq, Alexandre Persu, Nicholas Obermüller, Stefan W. Toennes

**Affiliations:** 1 Institute of Legal Medicine, Department of Forensic Toxicology, University Hospital, Goethe University, Frankfurt/Main, Germany; 2 Department of Nephrology, Medical Clinic III, University Hospital, Goethe University, Frankfurt/Main, Germany; 3 Division of Cardiology, Cliniques Universitaires Saint-Luc, Université Catholique de Louvain, Brussels, Belgium; 4 Clinical Chemistry Department, Cliniques Universitaires Saint-Luc, Brussels, Belgium; 5 Center for Toxicology and Applied Pharmacology, Université Catholique de Louvain, Brussels, Belgium; 6 Pole of Cardiovascular Research, Institut de Recherche Expérimentale et Clinique, Université Catholique de Louvain, Brussels, Belgium; International University of Health and Welfare, School of Medicine, JAPAN

## Abstract

With obesity having doubled in the last decade, hypertension is on the rise. In one-third of hypertensive patients the metabolic syndrome is present. This might be one factor for the increasing number of prescriptions for angiotensin receptor blockers and calcium-channel blockers besides a more favorable risk-to-benefit ratio. The aim of the present study was to evaluate a therapeutic drug monitoring (TDM) method for assessment of adherence based on cut-offs in inpatients and to compare it to an established urine drug screening in outpatients. A method for quantification of calcium-channel blockers and angiotensin receptor blockers using high-performance liquid chromatography-tandem mass spectrometric analysis (LC-MS/MS) was developed and validated. The method was applied to serum samples of 32 patients under supervised medication to establish cut-off values for adherence assessment based on dose-related concentrations (DRC, calculated from pharmacokinetic data). Furthermore, corresponding urine and blood samples of 42 outpatients without supervised medication were analysed and the results compared with regard to adherence assessment. All serum concentrations measured for amlodipine (n = 40), lercanidipine (n = 14), candesartan (n = 10), telmisartan (n = 4) and valsartan (n = 10) in inpatients were above the patient specific lower DRC confirming adherence. Of 42 outpatients the identification of analytes in urine as well as the quantification in serum exhibited differing results. According to urinalysis, adherence was demonstrated in only 87.0% of prescriptions, compared to 91.3% for serum analyses. Differences were observed for amlodipine, lercanidipine and candesartan which can be explained by a higher specificity of the serum analysis approach due to pharmacokinetics. The present study confirms that assessing adherence based on serum drug concentrations with individually calculated lower DRCs is more accurate than using qualitative urine analysis. In particular, drugs with low bioavailability, low renal excretion or high metabolism rate such as lercanidipine and candesartan may lead to underestimation of adherence via urine analysis.

## 1 Introduction

Hypertension is a widespread disease with a steadily increasing prevalence [1]. Since high blood pressure is one of the main causes of cardiovascular morbidity and mortality, the prevention and control of hypertension through lifestyle changes [1] and drug therapy is essential [2,3]. Still, despite the availability of efficient and generally well tolerated antihypertensive drugs, hypertension remains poorly controlled in a substantial proportion, if not the majority of hypertensive patients [4,5]. In a minority of patients, blood pressure remains uncontrolled despite prescription of three or more antihypertensive drug classes–so called resistant hypertension. It is generally accepted that one of the main factors leading to poor blood pressure control and apparent treatment-resistant hypertension (aTRH) is poor drug adherence [6]. This may be explained by the fact that high blood pressure causes few symptoms, especially in its initial stages, while the drugs have unpleasant adverse effects. In order to address this problem, improvement and wider availability of reliable methods for assessing patients’ adherence are required [7,8].

Since 2014, changes in prescription rates of antihypertensive drugs in Germany have occurred. An increase was noted for angiotensin receptor blockers (ARBs; + 24.8%) and calcium-channel blockers (CCBs; + 5.8%), whereas a decrease in prescription frequency was observed for ACE inhibitors (- 1.0%), β-blockers and diuretics (both—3.9%) [9,10]. This may be mostly related to a more favorable risk-to-benefit ratio and cost reduction of ARBs due to generics, but could also be explained to some extend by the rising prevalence of the metabolic syndrome, which is characterized by abdominal obesity, insulin resistance, hyperlipidemia and hypertension [11]. In this subset of patients, β-blockers and thiazide diuretics should be avoided if possible due to their diabetogenic effect [12].

For β-blockers and diuretics, a therapeutic drug monitoring (TDM) methodology and assessment of patient’s adherence based on individually calculated cut-offs has proved successful [13]. This concept utilized pharmacokinetic parameters to calculate cut-offs and covered differing dosage schemes. In view of the increasing prescription rates of angiotensin receptor blockers and calcium-channel blockers, the analytical method was extended by amlodipine, lercanidipine, candesartan, telmisartan and valsartan, which are the most frequently prescribed in Germany [9]. First, the method was evaluated with serum of hypertensive patients with confirmed adherence and then compared to an established urine drug screening with samples (urine, serum) of outpatients without supervised adherence to elucidate systematic differences in performance.

## 2 Materials and methods

### 2.1 Chemicals and reference standards

Reference substances of lercanidipine, telmisartan and valsartan were obtained from Sigma-Aldrich GmbH (Steinheim, Germany). Amlodipine and candesartan, as well as the deuterated internal standards (IS) diazepam-d_5_, lorazepam-d_4_ and nordiazepam-d_5_ were purchased from LGC Standards GmbH (Wesel, Germany).

Acetonitrile was obtained from Karl Roth GmbH (Karlsruhe, Germany) and ethyl acetate from AppliChem (Darmstadt, Germany). Further chemicals and solvents used were supplied by Sigma-Aldrich GmbH (Steinheim, Germany). All reagents and solvents were either of analytical or LC grade.

### Study design

#### 2.2.1 Inpatients with confirmed adherence

Patients (22 males, 10 females) aged 25 to 81 (median 59) years treated with a constant dosing regimen of antihypertensive drugs at the nephrological ward at the University Hospital Frankfurt/Main (Germany) participated in this study. Two blood samples were collected in serum tubes in the morning. The first one shortly before (trough level) and the second approximately two hours after monitored oral administration of the medication (peak level). After centrifugation (2,000 x g for 10 min) the separated serum was stored at -20 °C to ensure stability of the analytes. For data evaluation information on hospital admission, medication regimen (dose, dosing interval, date of last dose adjustment, co-medication), times of drug intake and of blood sampling were filed. Hospital admissions were documented to ensure that steady-state concentrations were reached by the time of blood sampling, and proper medication intake was monitored by the nurses.

#### 2.2.2 Outpatients

The study was carried out at the hypertension clinic of the Division of Cardiology, Cliniques Universitaires Saint-Luc (UCL, Brussels, Belgium). Patients (24 males, 18 females) included in this study were 36 to 79 (median 58) years of age and treated with in median 3 (range 1 to 7, inter-quartile range 2 to 4) different antihypertensive drugs. In contrast to the inpatients, their medication intake was not observed. Corresponding blood and urine samples for toxicological analyses were obtained at the same visit. After centrifugation (3500 x g for 10 min) the serum was separated. Thereafter, urine and serum samples were immediately frozen and stored at -20 °C until analysis to ensure stability. For data evaluation information on medication regimen (drugs, doses, dosing intervals), times of last scheduled drug intake and of blood and urine sampling were provided.

The study protocols were approved by the Ethics Committees of the University Hospital Frankfurt/Main and the Cliniques Universitaires Saint-Luc (Brussels). Both studies were in accordance with the 1964 Helsinki declaration and its later amendments. Written informed consent was obtained from all individual participants included in the studies.

### 2.3 Toxicological analysis of serum

The analysis procedure was essentially the same as described recently [13], but extended for detection of amlodipine, lercanidipine, valsartan, candesartan and telmisartan.

#### 2.3.1 Sample preparation

An aliquot of 200 μl serum was transferred to a 2 ml polypropylene reaction tube and 1 ml ethyl acetate, 50 μl internal standard working solution (0.5 ng/μl diazepam-d_5_, lorazepam-d_4_, and nordiazepam-d_5_ in acetonitrile), 10 μl of acetonitrile (or mixed standard solution or quality control mix, see below) and 50 μl formic acid (10%) were added. After mixing for 2 min and centrifugation at 13,000 x g for 10 min the organic phase was transferred to a silanized glass tube. Another 1 ml ethyl acetate and 50 μl of aqueous ammonia (25%) were added followed by mixing for 2 min, centrifugation and transferring to the glass tube. The combined extracts were evaporated at 25 °C with nitrogen using TurboVap LV (Biotage, Uppsala, Sweden). The dry residue was reconstituted with 100 μl of 0.1% formic acid/acetonitrile (80:20, v/v) and transferred to 300 μl glass vials of which 5 μl were analysed.

#### 2.3.2 Calibration standards and quality controls

Human drug-free serum for preparation of quality controls (QC) and calibration standards was provided by healthy volunteers. Stock solutions of lercanidipine and valsartan were prepared in acetonitrile, whereas amlodipine, candesartan and telmisartan were dissolved in methanol at concentrations of 1 mg/ml (drug) and kept refrigerated in darkness at -20 C, due to photosensitivity of calcium channel blockers [14]. These were used for preparation of mixed standard solutions and quality controls in acetonitrile. The calibration range was 0.05–10 ng/ml for lercanidipine, 0.5–100 ng/ml for amlodipine, 1.5–300 ng/ml for candesartan, 2–400 ng/ml for telmisartan and 40–8000 ng/ml for valsartan. The QC levels (low, medium, high) are given in Table 1.

**Table 1:** Validation data: Lower limit of quantification (LLOQ), limit of detection (LOD), intra- and inter-day precision, accuracy, recovery and matrix effects measured using the given quality control levels.

Analyte	LLOQ (LOD) [ng/ml]	quality control [ng/ml]	intra-day precision [%]	inter-day precision [%]	accuracy [%]	recovery [%]	Matrix effects ± SD [%]
Amlodipine	0.090	18.75	7.7	7.7	-4.6	77.7	94.3 ± 12.3
	(0.034)	62.5	8.1	8.7	0.2		
		87.5	5.2	7.2	0.5	89.0	122.1 ± 14.9
Lercanidipine	0.007	0.125	3.5	4.8	7.0	53.7	98.3 ± 3.5
	(0.001)	0.5	6.1	6.6	2.3		
		1.25	4.3	7.9	-1.5	78.4	109.8 ± 10.7
Candesartan	0.200	12.5	4.5	5.1	0.7	61.3	87.9 ± 8.9
	(0.080)	50	8.0	8.0	-0.8		
		125	2.8	4.8	1.3	86.9	91.7 ± 9.8
Telmisartan	0.004	12.5	4.4	4.4	3.0	55.0	97.0 ± 4.0
	(0.001)	50	5.9	5.9	0.7		
		125	4.1	4.6	-2.8	82.0	98.2 ± 7.3
Valsartan	0.487	125	6.2	7.1	-1.5	59.2	96.9 ± 8.8
	(0.250)	500	6.1	6.1	-2.5		
		1250	5.5	5.5	-2.1	88.8	88.2 ± 5.5

#### 2.3.3 Liquid chromatography-tandem mass spectrometry (LC-MS/MS)

The analysis was performed on an Agilent (Waldbronn, Germany) LC-MS/MS system consisting of a 1290 Infinity Liquid Chromatograph coupled via JetStream Electrospray Interface (ESI) to a G6460A Triple Quadrupole Mass Spectrometer. Extracts were kept at 20 °C on the autosampler and analytes were separated on a Kinetex^®^ 2.6 μm XB-C18 100 Å LC column (30 x 2.1 mm) plus corresponding guard column from Phenomenex (Aschaffenburg, Germany) at 55 °C.

Gradient elution was performed at a flow rate of 0.4 ml/min using 0.01% formic acid containing 5 mM ammonium formate (A) and acetonitrile containing 0.1% formic acid (B). Gradient elution started with 5% B kept for 0.5 min, increased to 40% B during 2.7 min, maintained 0.3 min, increased to 50% B within 0.5 min, held for 0.5 min and increased during 1.5 min to 95% B, maintained 2 min and followed by re-equilibration for 2 min, resulting in a total run time of 8 min.

Source parameters were selected as follows: gas temperature 300 °C, gas flow 11 l/min, nebulizer 45 psi, sheath gas temperature 400 °C, sheath gas flow 12 l/min and capillary voltage 3500 V. Detection was performed in the dynamic multiple reaction monitoring mode (dMRM, details given in Table 2). Data acquisition and evaluation was performed using Agilent MassHunter Software (version B.07.00). For identification of analytes a deviation of retention time of less than 0.05 min and a qualifier to quantifier ratio below 20% deviation compared to reference standards were required.

**Table 2:** Mass spectrometry parameters for the detection of calcium channel blockers and angiotensin receptor blockers using LC-MS/MS operated in dynamic MRM mode with two transitions for analytes and one for the corresponding internal standard. Retention times, MRM transitions and collision energies (CE) are given.

Analyte	Retention Time [min]	Precursor Ion [m/z]	Quantifier [m/z] (CE [eV])	Qualifier [m/z] (CE [eV])	Internal standard
Amlodipine	4.07	409.2	238.0 (4)	294.1 (4)	Lorazepam-d_4_
Candesartan	4.41	441.2	263.1 (4)	192.1(24)	Lorazepam-d_4_
Telmisartan	4.99	515.2	276.1 (48)	497.2 (32)	Diazepam-d_5_
Valsartan	5.01	436.2	291.1 (12)	235.1 (12)	Diazepam-d_5_
Lercanidipine	5.57	612.3	100.1 (36)	280.2 (20)	Nordiazepam-d_5_
**Internal Standard**					
Lorazepam-d_4_	4.31	325.1	279.1 (20)		
Nordiazepam-d_5_	4.62	276.1	140.0 (28)		
Diazepam-d_5_	5.12	290.1	198.1 (32)		

#### 2.3.4 Method validation

The method was validated according to current guidelines [15]. Statistical evaluation was performed with Valistat 2.0 Software (Arvecon GmbH, Walldorf, Germany).

In order to find appropriate internal standards for the analytes, 41 deuterated medical and illicit drugs were tested. Thus, a broad spectrum of substances with different chemical and chromatographic properties was evaluated. Internal standards were assigned regarding linearity and compensation of matrix effects. Retention time was the decisive factor if deuterated substances yielded similar results.

Matrix effects were evaluated by comparing peak areas of spiked extracts with those of standard solutions and recovery by comparing spiked matrix samples to spiked extracts. Both validation parameters were determined in low and high quality control samples. Each QC sample was measured six times using serum of different donors.

Selectivity was assessed with serum from eight different drug-free volunteers. Six samples were prepared without (blank samples) and another two by adding internal standard solution (zero samples). To show the absence of interferences, serum samples with exogenous substances including typical therapeutic drugs and metabolites, as well as a range of psychoactive substances were analyzed. Sensitivity was assessed by analysing five calibrator concentrations evenly spaced in the range of the expected limit of detection (LOD) and lower limit of quantification (LLOQ) as previously described [16].

Evaluation of linearity was done by six-fold determination of seven calibration levels evenly distributed across the calibration range. The calibration was checked for outliers (Grubbs test), homogeneity (Cochran test) and linearity (Mandel test).

For verification of accuracy and precision homogenous pools of low, medium and high quality control samples (relative to calibration range) were prepared by spiking blank matrix and dividing into aliquots. Thereafter, two quality controls of each concentration level were measured on eight different days. Results were tested for accuracy (bias ≤ 15%) and intra- and inter-day precision (relative standard deviation ≤ 15%).

Stability of extracted analytes was tested under autosampler conditions by repeated injection of an aliquot during 72 h. The analytes have been shown to be sufficiently stable during long-term storage and during freeze-thaw cycles [5,17,18].

### 2.4 Adherence assessment

#### 2.4.1 Serum samples

For evaluation of adherence the measured concentrations were compared with the lower limit of therapeutic reference ranges (TRR, literature data) [19–21] and with individually calculated lower limits of the dose-related concentration (DRC, calculated from data of pharmacokinetic studies), which are considered as promising cut-off for differentiating adherence from non-adherence as recently described by Ritscher et al. [13]. The dose-related concentrations include a diminution by one standard deviation of the apparent total clearance (lower DRC) to reflect interindividual variations in excretion. This can be individually calculated by multiplication of the patient’s drug dose with the drug’s lower DRC factor (Table 3) which is only valid for the given dosing interval (τ) and the time distances between last dose and blood sampling (Δt). Deviations in these parameters can be calculated using the two equations given in Table 3. For assessment of adherence, concentrations above these cut-offs (TRR, DRC) were considered as indicator of adherence to the respective drug.

**Table 3:** The data from pharmacokinetic studies refer to healthy volunteers (n = total number of volunteers) with data on bioavailability (f), apparent total clearance (CL_t_/f) and its standard deviation (SD), average elimination half-life (t_½_), the mean dose related concentration (DRC) factor with its lower limit for two time intervals between last dose and blood sampling (Δt). For other values of Δt the formulas below have to be employed. The last column cites the therapeutic reference range as retrieved from Schulz et al. [19].

Drug	N	f	τ [h]	CL_t_/f [ml/min] ^a^	SD [ml/min]	t_1/2_ [h]	Δt [h]	DRC factor ^c^[ng/ml/mg]	lower DRC factor ^d^ [ng/ml/mg]	reference	therapeutic range [ng/ml] [19]
Amlodipine	36	0.64	24 12	783.0 ^a^	361.1	37.0	2412	0.702	0.378	[25,30]	3–15
								0.791	0.426		
Lercanidipine	32	0.10	24 12	78988.9 ^a^	21787.5	3.4	24 12	0.002	0.001	[26,31]	1.2–13.6 [20]
								0.004	0.003		
Candesartan	30	0.15	24 12	1397.6 ^a^	325.2	11.6	2412	0.223	0.171	[27,32,33]	80–180
								0.340	0.261		
Telmisartan	51	0.50	24 12	1868.0 ^a^	872.4	24.6	2412	0.260	0.139	[21,34,35]	8.5–1299 [21] ^b^
								0.312	0.167		
Valsartan	36	0.24	24 12	465.7 ^a^	156.9	9.5	2412	0.549	0.364	[36,37]	800–6000
								0.933	0.619		

^a^ calculated by dividing the dose by the AUC

^b^ determined according to Consensus Guidelines for Therapeutic Drug Monitoring in Neuropsychopharmacology [23]

^c $DRC\;factor=\frac{f}{24*{CL}_{t}}*\frac{\tau *k_{e}}{1-e^{-k_{e}*\tau }}*e^{{-k}_{e}*\Delta t}[\frac{\frac{ng}{ml}}{mg}]$^

^d $lower\;DRC\;factor=DRC\;factor-(\frac{SD}{{CL}_{t}/f}*DRC\;factor)[\frac{ng}{ml}/mg]$^

#### 2.4.2 Urine samples

Analysis of urine samples and evaluation of results were performed as previously described [22]. For quality control, drug-free urine was used for preparing negative and positive control samples. The latter was spiked with all the reference substances. Analytical detection of a drug or metabolite in urine was considered an indicator of drug adherence.

## 3 Results

### 3.1 Method validation

The previously described method for quantification of β-blockers and diuretics was extended by five ARBs and CCBs. Blank and zero serum samples showed no significant interference in terms of endogenous substances at retention times of analytes or internal standards. Also, neither the spiked nor the analysed samples from patients exhibited interferences from other drugs.

**Fig 1:**
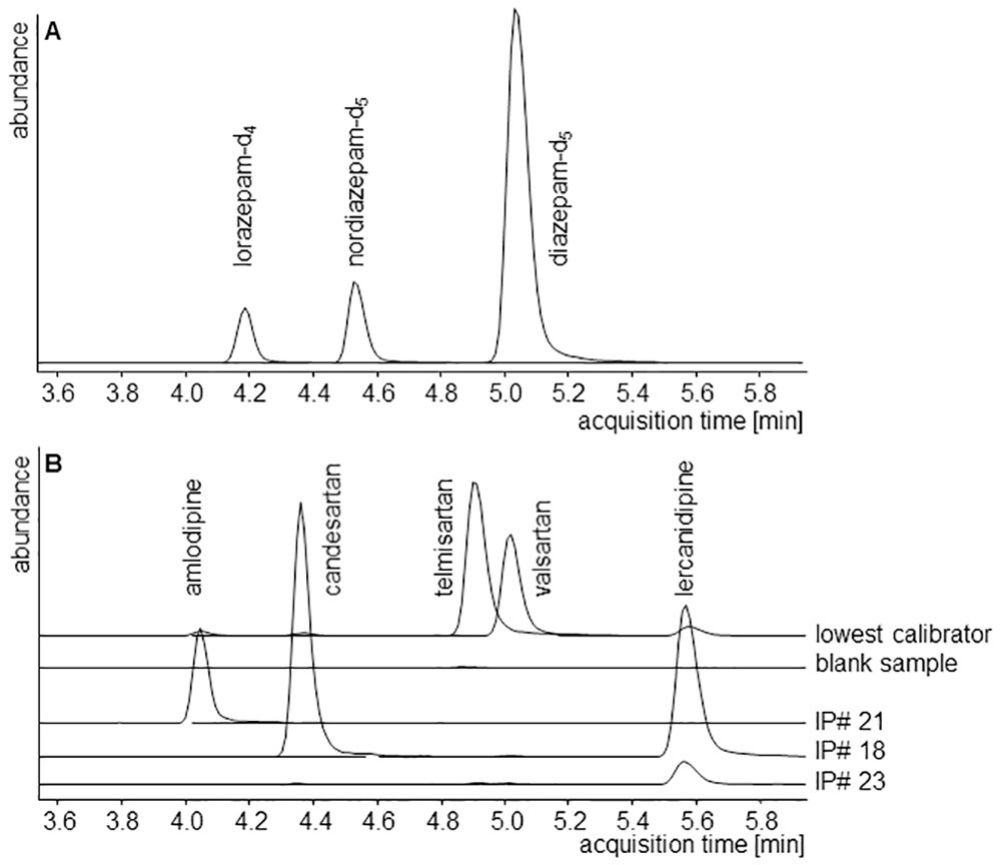
Representative extracted ion chromatograms of internal standards are given in (A) and of all analytes in the lowest calibrator, a blank sample, and trough serum samples of patient #IP 21 on amlodipine (14.8 ng/ml), patient #IP 18 on candesartan (72.0 ng/ml) and lercanidipine (3.2 ng/ml), and patient #IP 23 on lercanidipine (0.6 ng/ml) in (B). All signals are in equal scale.

Analyte signals were not affected by ion suppression and enhancement. The limit of detection, lower limit of quantification, intra- and inter-day precision, accuracy and recovery as part of the validation procedure are summarized (Table 1). The LLOQs were below expected serum concentrations. The requirements of the Grubbs test (95% significance level), Cochran test (99% significance) and Mandel test (99% significance) were fulfilled and a non-weighted calibration model excluding the origin was used. The calibration curves covered therapeutic ranges and were linear with regression coefficients of at least 0.999. Intra- and inter-day precisions were less than 8.7%, accuracies less than 7.0% (mostly < 3.0%) and recoveries higher than 50%. The concentrations of the extracted analytes decreased less than 25% during 72 hours of measurement, except of lercanidipine (48 h). Representative chromatograms are shown in Fig 1.

### 3.2 Inpatients with confirmed adherence

In this study arm the serum of 32 patients on antihypertensive medication were evaluated. All expected drugs could be quantitated (Table 4) where the trough levels before medication were of special interest. As expected, a marked increase in serum concentrations was observed in the serum samples about 2 hours after ingestion representing the time around peak concentrations.

**Table 4:** Concentrations of ARBs and CCBs in serum samples of inpatients (IP) shortly before and about 2 h after observed ingestion (trough / peak). Concentrations below or above published therapeutic reference ranges are indicated by “↓” or “↑”. No concentrations below the lower DRC (lower DRC factor * daily dose) were observed.

Patient #	Drug	Daily dose (single) [mg]	Amlodipine [ng/ml]	Lercanidipine [ng/ml]	Candesartan [ng/ml]	Telmisartan [ng/ml]	Valsartan [ng/ml]
IP 1	Amlodipine	10	8.3 / 10.5				
IP 2	Lercanidipine	20 (10)		1.2↓ / 4.8			
	Telmisartan	80 (40)				22.3 / 36.0	
IP 3	Amlodipine	2.5	7.5 / 8.6				
IP 4	Telmisartan	20				57.1 / 101.2	
IP 5	Amlodipine	10 (5)	13.8 / 21.3↑				
IP 6	Candesartan	8 (16)			6.0↓ / 56.3↓		
IP 7	Amlodipine	20	12.9 / 15.2↑				
	Valsartan	640 (320)					4570 / 7962↑
IP 8	Valsartan	40					115.5↓ / 384.8↓
IP 9	Amlodipine	10 (5)	8.8 / 11.2				
IP 10	Lercanidipine	10		2.2 / 5.5			
	Valsartan	320 (160)					5796 / 6040↑
IP 11	Amlodipine	10 (5)	8.5 / 10.0				
	Candesartan	32 (16)			153.6 / 246.7↑		
IP 12	Lercanidipine	10		0.2↓ / 1.8			
IP 13	Amlodipine	2.5	4.1 / 4.9				
IP 14	Amlodipine	5 (2.5)	2.4↓ / 2.9↓				
	Candesartan	16 (8)			91.8 / 170.2		
IP 15	Amlodipine	10	6.9 / 8.2				
IP 16	Valsartan	80 (40)					1827 / 2165
IP 17	Amlodipine	10	18.1↑ / 24.6↑				
IP 18	Candesartan	32 (16)			72.0↓ / 78.2↓		
	Lercanidipine	20 (10)		3.2 / 7.0			
IP 19	Valsartan	80 (40)					1007 / 1608
IP 20	Amlodipine	10 (5)	12.0 / 14.3				
IP 21	Amlodipine	10 (5)	14.8 / 18.3↑				
IP 22	Amlodipine	10 (5)	6.0 / 7.5				
	Candesartan	32 (16)			191.4↑ / 268.5↑		
IP 23	Lercanidipine	20 (10)		0.6↓ / 1.1↓			
IP 24	Amlodipine	10 (5)	31.7↑ / 26.7↑				
IP 25	Amlodipine	10 (5)	27.0↑ / 31.5↑				
IP 26	Amlodipine	10 (5)	26.1↑ / 35.8↑				
IP 27	Amlodipine	5	3.7 / 6.4				
IP 28	Amlodipine	10	19.3↑ / 24.4↑				
IP 29	Amlodipine	5 (2.5)	12.5 / 14.2				
IP 30	Lercanidipine	20		0.5↓ / 21.9^a^↑			
IP 31	Lercanidipine	20 (10)		1.1↓ / 6.1			
IP 32	Amlodipine	10 (5)	9.7 / 10.8				

^a^ above the highest calibration level

Reference data on therapeutic plasma levels were used from various sources [19–21] (Table 3). Most measured values were within the expected therapeutic reference ranges (57.7% of all determined concentrations). For amlodipine 32.5% of all measured concentrations exceeded the higher limit (> 15 ng/ml), as well as 30% of candesartan (> 180 ng/ml), 20% of valsartan (> 6000 ng/ml) and 7.1% of lercanidipine (> 13.6 ng/ml) serum levels. A high proportion of lercanidipine concentrations (42.9%) fell below the reported range (< 1.2 ng/ml), as well as 40% of candesartan (< 80 ng/ml), 20% of valsartan (< 800 ng/ml) and 5% of amlodipine (< 3 ng/ml) serum concentrations. In contrast, all determined concentrations of telmisartan were within the therapeutic reference range (8.5–1299 ng/ml) [21], estimated according to the procedure described in the Consensus Guidelines for Therapeutic Drug Monitoring in Neuropsychopharmacology [23].

In addition to evaluation of concentrations with regard to published reference ranges, the quantitative serum data was compared with the expected dose related lower limit of the trough serum concentration (lower DRC). This value was individually calculated on the basis of the patient’s drug dose and the drug’s lower DRC factor (Table 3). The lower DRC includes a diminution by one standard deviation of the apparent total clearance to reflect interindividual variations in excretion. All serum concentrations (trough and peak) of amlodipine, lercanidipine, candesartan, telmisartan and valsartan were above these calculated limits.

### 3.3 Outpatients

In this study arm, corresponding urine and serum samples of 42 patients on ARBs and CCBs were analysed and evaluated, including 27 patients treated with amlodipine, ten with lercanidipine, six with candesartan, one with telmisartan and two with valsartan. In total this were 46 drug determinations (prescriptions, drugs x patients).

#### 3.3.1 Urine

The results of urine analyses (Table 5) suggest adherence to telmisartan and valsartan in all patients. In two urine samples amlodipine was not found (7.4% of amlodipine treated patients), in three samples no lercanidipine was detected (30%) and in one sample the expected candesartan was absent (16.7%). Adherence to medication was assumed in 35 (85.4%) and non-adherence (one or more prescribed drugs not detected) in six patients (14.6%).

**Table 5:** Serum concentrations and qualitative results in urine from corresponding spot samples of outpatients (OP) regarding ARBs and CCBs. Prescribed dosages and times (Δt) between blood/urine sampling and last dose are given. Concentrations below or above published therapeutic reference ranges are indicated by “↓” or “↑”, concentrations below the lower DRC (c.f. [13]) are indicated by “*”. Positive results of the qualitative urinalysis are marked by “+”, negative results by “-”.

Patient #	Drug	Daily dose [mg]	Δ t [h]	Lower DRC [ng/ml]	Amlodipine[ng/ml]	Lercanidipine[ng/ml]	Candesartan[ng/ml]	Telmisartan[ng/ml]	Valsartan [ng/ml]
OP 1	Amlodipine	10	24	3,8	0.0↓* / -				
OP 2	Amlodipine	5	6	2,7	7.1 / +				
OP 3	Amlodipine	5	12	2,1	7.6 / +				
OP 4	Amlodipine	5	12	2,1	8.5 / +				
OP 5	Amlodipine	5	12	2,1	27.6↑ / +				
OP 6	Amlodipine	2.5	4	1,4	6.3 / +				
OP 7	Lercanidipine	10	16	0,03		1.0↓ / -			
OP 8	Telmisartan	80	2	20,4				375.5 / +	
OP 9	Lercanidipine	10	6	0,1		0.09↓* / -			
OP 10	Candesartan	16	4	9,0			108.7 / +		
OP 11	Amlodipine	10	6	5,3	10.5 / +				
OP 12	Amlodipine	10	6	5,3	8.0 / +				
OP 13	Amlodipine	5	6	2,7	5.7 / +				
OP 14	Amlodipine	5	24	1,9	12.2 / +				
	Candesartan	8	24	1,4			26.8↓ / -		
OP 15	Lercanidipine	20	24	0,02		18.7^a^↑ / +			
OP 16	Amlodipine	10	24	3,8	0.0↓* / -				
OP 17	Amlodipine	5	24	1,9	8.8 / +				
OP 18	Amlodipine	5	24	1,9	8.6 / +				
	Valsartan	120	24	43,7					3709 / +
OP 19	Candesartan	16	24	2,7			51.1↓ / +		
	Lercanidipine	20	24	0,02		6.1 / +			
OP 20	Amlodipine	10	24	3,8	13.2 / +				
OP 21	Amlodipine	5	24	1,9	6.0 / +				
OP 22	Lercanidipine	15	24	0,02		0.9↓ / +			
OP 23	Candesartan	8	20	1,7			6.6↓ / +		
OP 24	Amlodipine	5	6	2,7	4.1 / +				
OP 25	Amlodipine	10	14	4,6	15.7↑ / +				
OP 26	Lercanidipine	20	6	0,2		1.1↓ / +			
OP 27	Candesartan	16	6	8,0			70.0↓ / +		
OP 28	Amlodipine	5	2	2,9	4.7 / +				
OP 29	Lercanidipine	20	6	0,2		1.6 / +			
OP 30	Amlodipine	10	4	5,5	3.5* / +				
OP 31	Amlodipine	2.5	6	1,3	17.0↑ / +				
OP 32	Lercanidipine	20	14	0,1		1.5 / +			
OP 33	Amlodipine	5	8	2,6	8.2 / +				
OP 34	Amlodipine	5	16	2,2	8.6 / +				
	Valsartan	160	16	104,3					2306 / +
OP 35	Lercanidipine	20	24	0,02		1.0↓ / -			
OP 36	Candesartan	20	24	3,4			5.8↓ / +		
OP 37	Amlodipine	5	24	1,9	7.2 / +				
OP 38	Amlodipine	10	24	3,8	7.2 / +				
OP 39	Lercanidipine	20	24	0,02		0.9↓ / +			
OP 40	Amlodipine	10	24	3,8	18.3↑ / +				
OP 41	Amlodipine	10	24	3,8	11.8 / +				
OP 42	Amlodipine	5	24	1,9	22.6↑ / +				

^a^ above the highest calibration level

#### 3.3.2 Serum

Of the 42 patients 27 were treated with amlodipine (4.1 to 27.6, median 8.5 ng/ml), ten with lercanidipine (0.85 to 18.7, median 1.1 ng/ml), six with candesartan (5.8 to 108.7, median 39.0 ng/ml), one with telmisartan (375.5 ng/ml) and two with valsartan (2306 to 3709, median 3007 ng/ml). These concentrations are in accordance with those that were measured in the patients with confirmed adherence (see Table 4) and with literature.

For evaluation, the quantitative serum data were compared with the individually calculated lower DRCs. All measured serum concentrations of patients on candesartan, telmisartan and valsartan were above the respective calculated limits as indicator of medication adherence. However, the fraction of patients which exceeded the lower DRC was 90% for lercanidipine and 88.9% for amlodipine. In combination, 38 patients (90.5%) would be considered as adherent to their medication.

In addition to the above evaluation approach published therapeutic ranges were also compared to the determined serum concentrations (Table 3). Using the lower limit of the therapeutic reference range as cut-off, patients’ adherence to the measured drugs would be assumed in 33 (78.6%) of the 42 patients (Table 5).

#### 3.3.3 Urine vs serum samples

In terms of adherence assessment, the qualitative urine screening results agreed with the quantitative serum assays for telmisartan and valsartan. However, this does not apply to amlodipine, lercanidipine and candesartan. Three patients on lercanidipine and one patient on candesartan were rated as non-adherent according to the urine screening, whereas the quantitative results in serum suggested adherence. In contrast, one patient on amlodipine was classified as adherent in urine and as non-adherent in serum.

## 4 Discussion

Hypertension incidence is on the rise which is not surprising with the prevalence of obesity having more than doubled in the last three decades [11]. The current recommendations on pharmacotherapy favor the use of ARBs and CCBs due to their more favorable risk-to-benefit ratio in terms of tolerability (especially ARBs) and prevention of organ damage [24]. Considering this and in order to evaluate the potential of misinterpretation using qualitative methods, a quantitative serum assay for ARBs and CCBs was developed as recently published for β-blockers and diuretics [13]. Individually calculated cut-offs were used for classification of adherence. In a first study arm the reliability was evaluated in a number of patients with supervised medication adherence. In a second study arm this method was applied to blood samples from outpatients who also provided urine samples allowing the comparison of the performance of both methods.

### 4.1 Serum concentrations

#### 4.1.1 Patients with confirmed adherence

Of the 32 patients 20 were treated with amlodipine, which was confirmed in concentrations (trough and peak) of 2.4 to 35.8 ng/ml, seven with lercanidipine (0.24 to 21.9 ng/ml), five with candesartan (6.0 to 268.6 ng/ml), two with telmisartan (22.3 to 101.2 ng/ml) and five with valsartan (115.5 to 7962 ng/ml). The measured concentration ranges are in accordance with those found in samples of a routine TDM [5] for lercanidipine (0.07–10.5 ng/ml), candesartan (7.05–440 ng/ml) and valsartan (30.9–2613 ng/ml). However, no data on daily doses or times of blood sampling was provided in that report for a more detailed comparison. The larger concentration ranges in the present data are in agreement with the sampling scheme targeting the minimal (trough) and maximal (peak) concentrations in the patients. In contrast, the upper limit of the concentration range reported in this routine TDM setting by Gundersen et al. [5] appears rather high for amlodipine (5.44–126 ng/ml). The results of a double-blind placebo-controlled study in 56 healthy volunteers receiving repeated oral doses of amlodipine 15 mg (off-label use, regular doses: 5–10 mg) once daily exhibited trough concentration of 11.8 ± 5.3 ng/ml and peak concentrations of 18.1 ± 7.1 ng/ml at steady-state [25]. This is in agreement with the concentrations found in this study. The present study results also match reported serum concentrations of telmisartan. In a randomized, double-blind, placebo-controlled study, concentrations in blood of patients receiving repeated oral doses of telmisartan ranged from 47.0 ± 38.5 ng/ml (trough concentration with the dose of 40 mg/d, n = 39) up to 693 ± 606 ng/ml (peak concentration with the dose of 80 mg/d, n = 37) [21]. For lercanidipine, in one patient a remarkably high serum concentration was measured (21.9 ng/ml, Table 4, #IP 30). This may be explained by an increased oral bioavailability in conjunction with a high fat-meal, which is assumed due to the marked milky turbidity of this sample. In such cases a threefold increase in plasma concentrations has been described [26].

#### 4.1.2 Outpatients

The 42 outpatients were in median treated with three different drugs. Since the analyses only looked at ARBs and CCBs, adherence rates referring to individual patients should not be taken as representative for adherence to all drugs of the medication regimen.

The concentrations measured above the individually calculated lower DRCs (Table 5) agree with the results of the inpatients with assured adherence (4.1.1) and with concentration ranges reported in other studies [5,21,25]

### 4.2 Assessment of adherence

Adherence assessment is important to distinguish between true treatment resistance and apparent treatment resistant hypertension (aTRH) due to non-adherence. Several methods for assessing patients’ adherence have been published. Recently, a methodology employing quantitative data in combination with cut-off values was published. This method was initially evaluated for diuretics and β-blockers only. Since diuretics and β-blockers are no longer considered as the first-line treatment in the majority of hypertensive patients [24], this method was extended to calcium-channel blockers and angiotensin receptor blockers.

#### 4.2.1 Patients with confirmed adherence

The group of patients with confirmed adherence were used for evaluation of the analytical method and the reliability of using the lower DRC as cut-off to diagnose therapeutic concentrations.

The comparison of measured concentrations with published reference ranges exhibited marked deviations, which was expected from previous experiences [13]. Of the 78 trough and peak concentrations measured 19 (24.4%, 33.3% of the 39 peak values only) exceeded the upper therapeutic limit. This mainly affected patients (8 patients, 13 values) receiving a rather high daily dose of amlodipine (7 patients with 10 mg and one patient with 20 mg). On the other hand, 14 values fell below the lower limit of the published concentration range, this particularly affected lercanidipine (6 values) and candesartan (4 values). The reference ranges as tabulated in Table 3 obviously do not cover concentrations resulting from rather low and high doses and are therefore not reliable for classification with regard to adherence assessment.

In contrast, all measured values were above the calculated minimum concentrations expected for the respective dosage schemes (lower DRC). This confirms the superiority of this evaluation concept which has also been shown for TDM in neuropsychopharmacology [10] and for β-blockers and diuretics [13].

#### 4.2.2 Assessment of adherence in outpatients on the basis of quantitative serum analysis results

The determined values were compared with the lower limit of concentration ranges considered therapeutic (Table 3) but for differentiation of adherence only the lower DRC was used as cut-off. This value is calculated individually for different dosing regimens based on the concept of Hiemke et al. [13,23]. Overall, four of 46 determined drug concentrations (8.7%) were beneath the lower limit of the dose-related concentration range which indicates that the respective drug (amlodipine n = 3, lercanidipine n = 1) was not ingested as required (non-adherence) or that the subject exhibited a deviation in pharmacokinetics (e.g. malabsorption or rapid drug elimination).

#### 4.2.3 Assessment of adherence in outpatients on the basis of qualitative urine analysis results

In the present group of outpatients, adherence rates for ARBs (88.9%) and CCBs (86.5%) according to detection in urine samples were high. This is in accordance with results of previous studies using qualitative toxicological analysis to assess adherence [22]. Overall, in 6 of 42 patients (14.3%) non-adherence to medication with amlodipine (n = 2), lercanidipine (n = 3) and candesartan (n = 1) would have been assumed.

### 4.3 Comparison of adherence assessment using urine or serum analysis results

Adherence rates differ between classes of antihypertensive medications and between different methods of adherence assessment. Apart from indirect methods such as self-reporting, pill counting or electronic monitoring systems, qualitative analysis methods may also be imprecise. This would be the case if urine analysis still yields a positive result even if some time passed since the last drug ingestion, which can be due to the high sensitivity of the applied analytical method or a long excretion of drugs. This may lead to an overestimation of adherence (e.g. HCT [13]) but also an underestimation is possible, e.g. in case of analytes which are highly metabolised (e.g. nebivolol [13]) and hardly excreted via the kidneys. Therefore, the current study was performed to evaluate a more sophisticated and improved method to detect “pseudo-resistant hypertension” caused by non-adherence to ARBs and CCBs.

**Fig 2:**
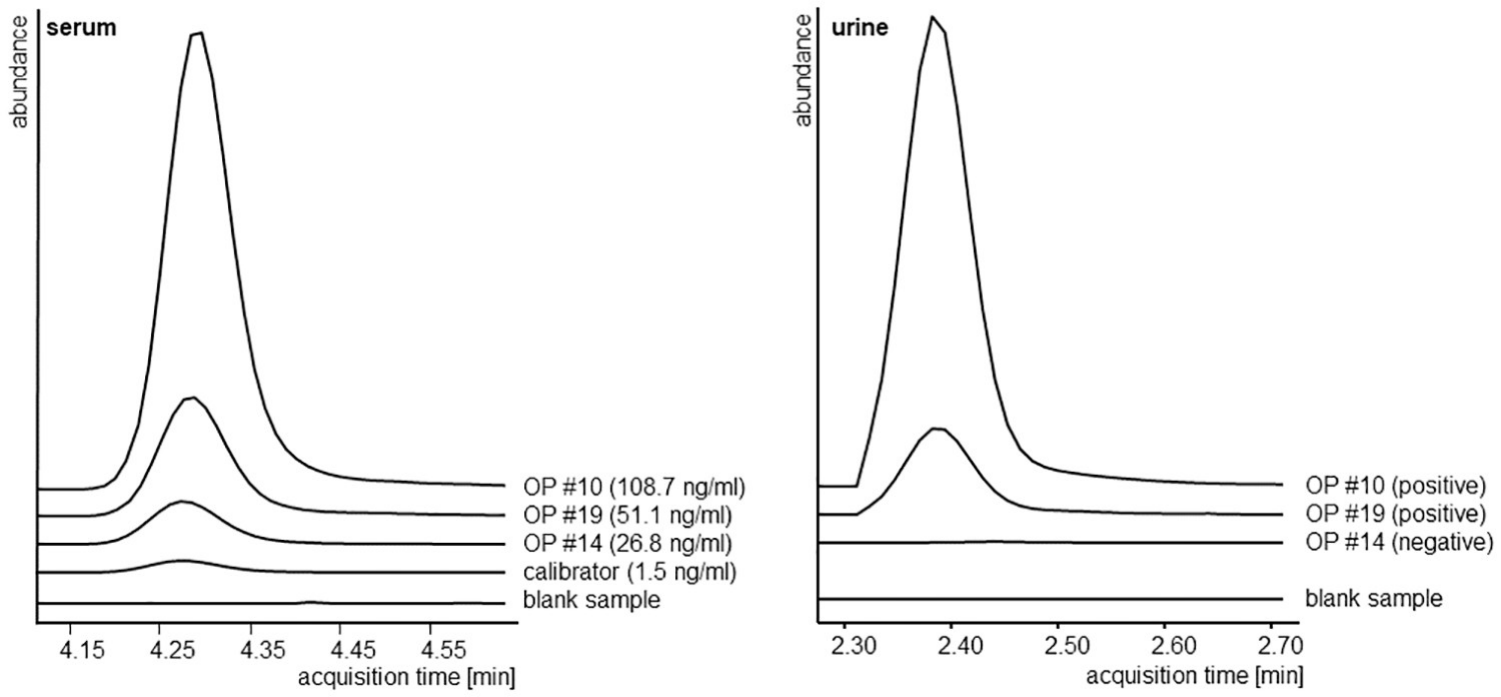
Analytical data (extracted ion chromatograms in equal scale) of candesartan in serum and urine. Patient #OP 14 with a serum concentration above the respective DRC (1.4 ng/ml) but a negative urine analysis result, in contrast to results in two representative patients (#OP 10 and #OP 19) being positive in serum and urine.

The results in outpatients show that urine and serum findings differ for amlodipine, lercanidipine and candesartan medication. In one serum sample (#OP 30, Table 5) the amlodipine concentration was markedly beneath the lower DRC, where, on a sole qualitative basis, adherence would have been assumed. Lercanidipine was not detected in three of ten (30%) urine samples (patients #OP 6, #OP 9 and #OP 35), which would result in classification of these patients as non-adherent for this drug. In contrast, the serum concentrations of patients #OP 6 and #OP 35 were above the lower DRC (Table 5) which suggests sufficient adherence. Only patient #OP 9 would be classified as non-adherent by both methods. This discrepancy can be explained by an extensive first-pass metabolism of orally administered lercanidipine by cytochrome P450 3A4 [26]. Almost no unchanged drug is eliminated renally and might therefore not be detected in urine assays though the analytical sensitivity is high enough to detect it in a number of cases. For candesartan one urine sample was negative (patient #OP 14, Fig 2) while serum analysis suggested sufficient adherence. A lack of detection in urine might again be due to the low bioavailability of candesartan of only 15% after oral administration of its prodrug candesartan cilexetil [27]. Of this bioavailable dose only about 26% is excreted unchanged via the kidneys with a low renal clearance [27]. This might be responsible for false negative urine results.

Though the applied analytical method for urine analysis is highly sensitive and able to detect lercanidipine as well as candesartan in patients’ urine samples [22] the results of patient #OP 6, #OP 14 and #OP 35 suggest a limitation. The comparison of the two methods shows that according to serum analysis only four patients would be classified as non-adherent to at least one drug while with the qualitative urine method this were six patients. An improvement of the urine method could be the inclusion of further metabolites [28], but the present approach of evaluating serum concentrations appears superior in targeting the specific active drugs and may lead to a better assessment of drug adherence in general. However, it should be kept in mind that adherence may considerably vary over time [29] which is not reflected by direct methods.

## 5 Conclusion

The present study was performed to evaluate assessment of adherence to angiotensin receptor blockers and calcium-channel blockers based on quantitative serum drug concentrations using lower dose-related concentration as cut-off values. The results with patients on confirmed medication adherence show that this concept is more promising in contrast to using published ranges of therapeutic concentrations.

In a group of outpatients without supervised adherence the quantitative serum analysis method was compared to a qualitative urine assay. The results show that drugs with low bioavailability, low renal excretion or high metabolism rate may lead to underestimation of adherence via urine analysis. Using individually calculated lower DRCs as cut-off for serum concentrations is suggested as a more appropriate method for adherence assessment.
